# AistSeq: An in-house easy-to-purify Tn5-based plasmid sequencing platform using a compact benchtop sequencer

**DOI:** 10.3389/fbioe.2025.1673510

**Published:** 2026-01-27

**Authors:** Hayato Suzuki, Jutapat Romsuk, Akiyoshi Nakamura, Kosuke Moriwaki, Yoko Yamanishi, Shigeo S. Sugano

**Affiliations:** 1 Plant Molecular Production Research Team, Biomanufacturing Process Research Center, The National Institute of Advanced Industrial Science and Technology (AIST), Sapporo, Japan; 2 Plant Functional Regulation Research Team, Biomanufacturing Process Research Center, The National Institute of Advanced Industrial Science and Technology (AIST), Tsukuba, Japan; 3 Department of Mechanical Engineering, Kyushu University, Fukuoka, Japan

**Keywords:** bench top sequencer, next-generation sequencing (NGS), pipeline, plasmid sequencing, Tn5 transposase purification

## Abstract

Sequence verification of plasmids is a fundamental process in synthetic biology. For plasmid sequence verification using next-generation sequencing (NGS) library preparation, Tn5 transposase is widely used. Streamlined sequencing workflow for laboratory-scale applications is important; however, recombinant Tn5 production in-house can be laborious. In this study, we demonstrated that the addition of a large soluble tag was not essential for purification and that the fusion of a His10 tag and protein A was sufficient to yield active Tn5 transposase in adequate amounts. In addition, we evaluated exonuclease-based genomic DNA digestion for plasmid sequencing from an *E. coli* lysate and the data analysis pipeline of sequences derived from the Illumina iSeq100 platform for *de novo* assembly, reference mapping, and annotation. This study proposes a simple workflow of an in-house easy-to-purify Tn5-based plasmid sequencing platform using a compact benchtop sequencer (AistSeq).

## Introduction

Tn5 transposase, discovered in the early 1990s in *Escherichia coli* (Blot et al., 1993), is pivotal for next-generation sequencing (NGS) library preparation. It forms a ternary complex known as the Tn5 transposome, which consists of a homodimer of the Tn5 protein and its DNA cargo. This offers versatility in custom DNA and adapter payloads (Li et al., 2020). The Tn5 transposome can fragment DNAs and attach sequencing adapters simultaneously, thus streamlining NGS workflows, such as the Nextera library preparation kit (Caruccio, 2011) and ATAC-Seq (Buenrostro et al., 2013). Plasmid tagmentation using Tn5 protein followed by long-read sequencing with Oxford Nanopore has also been reported (Emiliani et al., 2022). Recent studies have demonstrated the ability of Tn5 to target DNA/RNA hybrids, thereby broadening its applications to RNA sequencing (RNA-seq; Di et al., 2020); however, recombinant Tn5 transposase is not so easy to produce in the laboratory scale. There are several methods for obtaining sufficient purified Tn5, including TnY (Hennig et al., 2018; Liscovitch-Brauer et al., 2021; Xu et al., 2021), however, the fusion of large soluble tags or chitin-binding protein tags to the transposons is necessary. Recently, OpenTn5 open-source and easy-to-purify Tn5 was proposed (Soroczynski et al., 2024), applicability of this methodology has not been explored.

These developments have addressed some of the challenges faced by industrial-scale synthetic biologists, such as the high costs associated with Sanger sequencing for plasmid verification. Integrating Tn5 into plasmid sequencing workflows drastically reduces costs, thus enabling sequencing of over 4,000 plasmids in a single Illumina MiSeq run for <$3 sequencing each (Hwang et al., 2019; Shapland et al., 2015). To minimize preparation costs, plasmid sequencing from an *E. coli* lysate without purifications has been demonstrated (Hwang et al., 2019). In addition, various software programs and pipelines have been developed to support and verify plasmid sequencing analysis (Hwang et al., 2019). Likewise, there is a significant demand for streamlined plasmid sequencing methods utilizing Tn5 transposome. Here, we propose an alternative approach to Tn5 transposase purification, in which only a His10 tag and protein A (pA) are used to purify Tn5 transposase. In addition, we developed a simple in-house workflow for plasmid-DNA sequencing of *E. coli.* lysates by Illumina iSeq100 platform. We also established an alternative bioinformatics pipeline to accommodate *de novo* sequence assembly and alignment with reference plasmid sequences for generating a FASTA file comprising the sequence of the plasmid and an annotated map, encompassing insertion, point mutations, and deletions to the reference. This streamlined workflow for the verification of plasmid-DNA sequences would contribute to molecular biology including the synthetic biology field.

## Results and discussion

### *In silico* analysis pipeline

**FIGURE 1:**
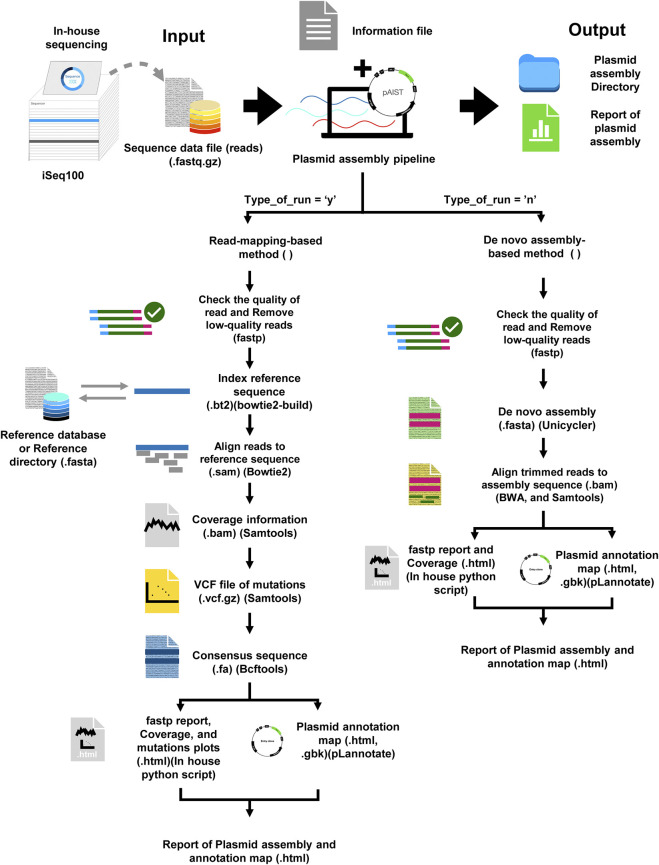
Workflow of the bioinformatic pipeline for plasmid sequencing developed in this study. The pipeline automates plasmid sequence analysis from paired-end FASTQ reads (.fastq.gz) using either a reference-mapping-based workflow or a *de novo* assembly approach. Input data include sequencing reads and a user-defined information file specifying run parameters and optional reference sequences. For reference-based runs (Type_of_run = “y”), reads are quality-filtered with *fastp*, aligned to the reference using *Bowtie2*, and processed with *Samtools* and *Bcftools* to generate coverage statistics (.bam), mutations (.vcf.gz), and a consensus sequence (.fasta). For *de novo* assembly (Type_of_run = “n”), trimmed reads are assembled using *Unicycler*, followed by read mapping with *BWA* for coverage assessment (.bam). In both workflows, the final sequence is annotated using pLannotate to produce GenBank output (.gbk) and an interactive plasmid map (.html). The pipeline generates individual HTML reports summarizing quality metrics, coverage, and assembly results for each plasmid sample. Furthermore, an additional summary report (.html) provides an overview of run times and the success of plasmid reconstruction for all processed samples.

We developed a comprehensive bioinformatics pipeline for the analysis and reporting of plasmid sequencing obtained from iSeq100 paired-end 150 bp data (Figure 1). This program offers automating *de novo* assembly, mapping to a reference sequence, and showing several metrics (Supplementary Figures 1a,b). *De novo* assembly of plasmid was conducted using Unicycler (Hwang et al., 2019), originally proposed as a *de novo* assembly pipeline for bacterial genomes. Mapping to the reference sequences was performed using Bowtie2 (Langmead and Salzberg, 2012). For mapping results, consensus sequences were outputted as “.fasta” files and were accompanied by coverage plots for each plasmid (Supplementary Figure 1c). Mutated nucleotide positions (Supplementary Figure 1d) were identified by bcftools (Danecek et al., 2021) and BEDtools (Quinlan and Hall, 2010). The output consensus sequences were automatically annotated by pLannotate (McGuffie and Barrick, 2021) to enhance user-friendliness (Figure 1; Supplementary Figure 1e).

Here, by combining this analysis pipeline with the simple pA-Tn5 purification and library preparation using *E. coli* lysate supernatant, we provide a streamlined plasmid verification workflow, an in-house Tn5-based plasmid sequencing platform using a compact benchtop sequencer, designated AistSeq.

### Expression of His10-pA-Tn5 is sufficient to purify abundant Tn5 proteins

**FIGURE 2:**
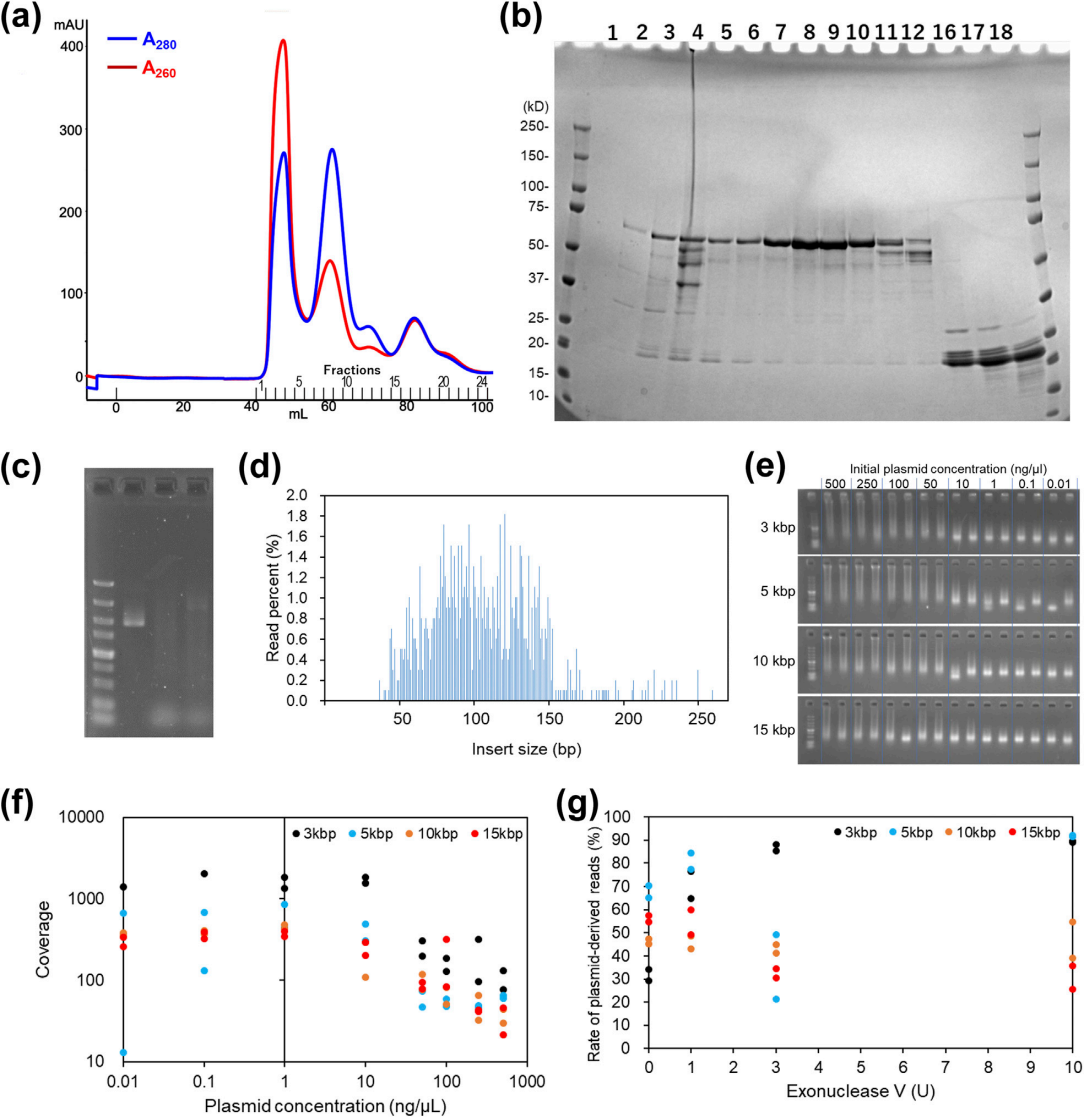
Tn5 transposon purification, Tn5-based plasmid digestion, and output of NGS analysis of plasmid prepared by several methodologies. Size exclusion chromatogram **(a)** and SDS-PAGE **(b)** to assess the purity of Tn5 transposase. **(c)** Gel electrophoresis of intact pUC18 plasmid (left), digested by commercial Tn5 (diagenode; middle) and digested by in-house His10-pA-Tn5 (right). **(d)** Size distribution of sequencing inserts confirmed by iSeq100 sequencing. **(e)** Tn5 digestion of plasmids with different lengths and concentrations. **(f)** Sequencing coverage plots for the NGS analysis of plasmids with different lengths and concentrations. **(g)** The ratio of plasmid-derived reads to total reads in the libraries constructed from the supernatant of *E. coli* lysates incubated with various concentrations of ExoV. **(e–g)** Experiments were performed in duplicate (technical replicates, *n* = 2).

Recombinant Tn5 transposase tightly binds to nucleic acids during the purification step (Buenrostro et al., 2013; Caruccio, 2011; Xu et al., 2021). Although a chitin column was used for the purification of Tn5 transposase, a recent report revealed that GB1-Tn5 transposase with a His10-tag can be purified free from nucleic acid contamination by washing with a high salt buffer (800 mM NaCl) on a Ni-affinity column (Xu et al., 2021). We constructed a His10-tagged pA-Tn5 expression vector, which is a smaller tag compared with GB1, and purified recombinant pA-Tn5 using a Ni-affinity column. Recently reported OpenTn5 method is like our approach that it takes advantage of protein G instead of pA (Soroczynski et al., 2024). Following Ni-affinity purification, recombinant pA-Tn5 transposase was further purified by size exclusion chromatography. The peaks from size exclusion chromatography (Figure 2a) and SDS-PAGE analysis of the eluted fractions (Figure 2b) suggested that the majority of pA-Tn5 transposase dimer did not bind nucleic acids, which was confirmed by measuring the 260 nm/280 nm ratio. The dimer fractions containing pA-Tn5 transposase were collected and concentrated to 60 µM. The purified pA-Tn5 assembled to oligonucleotides (harboring mosaic end sequences with Illumina adapter sequences) successfully fragmented pUC18 plasmids, like commercially available Tn5 (Figure 2c), which was also supported by the results of iSeq100 sequencing (Figure 2d).

In the purification of Tn5 protein from *E. coli*, size exclusion chromatography is indispensable for reducing contamination from endogenous genomic DNA (Figure 2a). As discussed by Soroczynski et al. (2024), omission of this step may also compromise protein stability due to aggregation. Nevertheless, further optimization of the Ni-affinity purification conditions to enhance purity would provide a more streamlined purification workflow, representing an interesting direction for future improvement.

Compared with Sanger sequencing, which typically costs $3 for sequencing 500–1,000 bp, whole plasmid sequencing using Tn5 and NGS offers a substantial cost advantage (Hwang et al., 2019). In this workflow—comprising plasmid extraction, Tn5-based library preparation, and sequencing with the iSeq100 system—the Tn5 enzyme accounts for approximately 46% of the total cost (Supplementary Table 1). From 1 L of *E. coli* culture, 3.25 mg of Tn5 protein was obtained, sufficient for approximately 64,000 reactions. Using commercial Tn5 for plasmid extraction, library construction, and sequencing with the iSeq100 system cost $6.32 per plasmid, whereas use of our in-house Tn5 reduced the cost to $3.37 per plasmid, highlighting its clear economic advantage. In the OpenTn5 paper (Soroczynski et al., 2024), approximately 3 mg of pG-Tn5 was obtained from 500 mL of *E. coli* culture. Their protocol included several optimizations, such as the use of Terrific Broth (TB) instead of LB medium and the addition of an anti-foaming agent during cultivation. Similar modifications could potentially further improve the yield of pA-Tn5 and reduce the costs in our system.

### Tn5-based whole plasmid sequencing and the plasmid size/concentration dependency

Tagmentation of 3, 5, 10, and 15 kb plasmids at concentrations ranging from 0.01 to 500 ng/μL was evaluated using in-house-prepared pA-Tn5. The Gel electrophoresis of the PCR products revealed that higher initial plasmid concentrations resulted in DNA libraries with larger average fragment sizes (Figure 2e). The sequencing coverage was lower at higher initial plasmid concentrations (Figure 2f), which was attributed to insufficient Tn5 proteins resulting from excess plasmid and predominantly larger DNA fragments. Thus, maintaining an appropriate Tn5:plasmid ratio is necessary for DNA library preparation. For larger plasmids (10 and 15 kbp), diluted plasmids (<10 ng/μL) are preferable.

### Sequencing plasmid from *E. coli* lysates and assessing genome contamination

Direct NGS library preparation of the plasmid to be sequenced from *E. coli* lysates offers time and cost savings; however, *E. coli* genome contamination can hinder plasmid-derived read recovery (Hwang et al., 2019). We evaluated exonuclease V (ExoV) efficacy in selectively digesting damaged genomes before library preparation because circular plasmids without ends are not digested by ExoV. ExoV (0, 1, 3, and 10 U)-treated lysates of *E. coli* harboring high-copy plasmids of various lengths (3, 5, 10, and 15 kb) were assessed after the separation of the lysate pellets and supernatants (Supplementary Table 2). The lysates had higher genomic DNA content, which impaired plasmid fragmentation and reduced read quality compared with the supernatants. Using the pellet samples, full-length plasmid sequences were obtained for 3 and 5 kb plasmids, but larger plasmids (10 and 15 kb) were incompletely assembled. Higher ExoV concentrations negatively affected sequencing results for larger plasmids (supernatant samples), which was likely caused by susceptibility to linearization of plasmid during vortexing and pipetting (Figure 2g). Remarkably, assembly into one circular sequence was successfully achieved in supernatant samples treated with 0 or 1 U of ExoV, whereas the pellet samples yielded few successful assemblies through *de novo* assembly (Supplementary Table 2). These findings suggest the universal suitability of supernatant samples treated with 0 or 1 U ExoV.

As a result of the *de novo* assembly, 41 out of 64 samples (64%) produced circular sequences. Among all samples, 21 yielded assemblies containing multiple contigs. The linear contigs were derived from incomplete plasmid assemblies or *E. coli* genomic fragments. In reference mapping, consensus sequences were successfully generated for 57 samples (89%) with sufficient read depth, and the assembly error rates were below 0.005%, providing more stable results than those obtained by *de novo* assembly (Supplementary Table 2).

In synthetic biology, low-copy plasmids are often used as gene constructs. To evaluate whether our method can also be applied to such plasmids, we transformed *E. coli* DH5α, HST08, and HST16CR (Takara Bio) with a pUC18-derived plasmid and performed library preparation and sequencing directly from the lysates. DH5α and HST08 maintain pUC18-derived plasmids at high copy numbers, whereas HST16CR, which lacks pcnB, maintains them at low copy numbers. The supernatant samples were treated with 1 U of ExoV and used for library preparation. Approximately 60,000 reads were sequenced, which is considered excessive for plasmid sequencing. In the cases of DH5α and HST08, about 80% of the reads were derived from plasmids. However, in HST16CR, only around 0.5% of the reads mapped to plasmids, with most mapping to the *E. coli* genome, making it difficult to obtain a sufficient number of reads for plasmid sequence reconstitution (Supplementary Table 3). This indicates a technical limitation of our method, suggesting that purified plasmid DNA should be used for sequencing analysis of low-copy plasmids. The activity of ExoV is known to be affected by the type and concentration of cations (e.g., Mg^2+^ and Ca^2+^), and vortexing can influence plasmid shearing. Optimization of these parameters may help prevent unintended plasmid linearization and improve sequencing outcomes.

### Application to long and complex plasmids and multiplex sample analysis

Plasmids exceeding 15 kbp are frequently constructed, and the ability to analyze such large plasmids is essential for assessing the practical utility of AistSeq. In ExoV-treated *E. coli* lysates, however, plasmids of 10–15 kbp and low-copy plasmids showed unstable results in both *de novo* assembly and reference mapping. We therefore applied AistSeq to purified plasmids with 15–20 kbp in length (Supplementary Table 4). *De novo* assembly yielded circular sequences for 4 of the 16 constructs, whereas reference mapping successfully generated consensus sequences for all constructs.

In plant transformation, binary vectors often carry multiple expression cassettes within the Transfer-DNA region, resulting in duplicated promoters, terminators, and other elements. These repeated sequences further hinder circular assembly using *de novo* methods. Notably, VB0060 (pGWB17), an approximately 17 kbp plasmid containing two cauliflower mosaic virus 35S promoters and three nopaline synthase (NOS) terminators, was accurately reconstructed by reference mapping (Supplementary Table 4).

AistSeq is a streamlined platform to determine a large number of plasmids simultaneously. In our laboratory, samples were pooled to obtain approximately 5,000–10,000 reads per plasmid, allowing us to analyze up to 384 plasmid sequences in a single iSeq100 run.

## Materials and methods

### Bioinformatics pipeline for plasmid assembly and comprehensive analysis

We implemented a script to encompass several key analysis tools and steps, each contributing to the overall process of automating the *de novo* assembly of plasmid, mapping to reference, and subsequent analysis. The script begins with a definition of the two essential functions that are responsible for handling plasmid assembly with or without providing reference sequence data. Using command line arguments, the script designates input and output directories, reference databases, and the input samplesheet file. Next, it scans the command line parameters to determine the input.zip file and the text file that contains relevant analysis details. To run the script, users must prepare their input data and execute the script in a Linux-based environment. This involves establishing a directory to organize the input data, placing FASTQ files and a text file with the analysis information in this directory, adjusting the settings to their computing environment, and executing the script from the command line. The FASTQ files (read) in the directory are named according to the information in the text file used for the input. The information in the text file (.txt) contains information regarding user analysis. Each line of the text file represents a different analysis (sample) and should include the following columns (Tab-separated values):

1. Primary: The attributes used to identify a record.
2. read1: The name of the FASTQ file (. fastq.gz) containing the first set of short reads in each pair.
3. read2: The name of the FASTQ file (. fastq.gz) containing the second set of short reads in each pair.
4. expected_reads: The expected number of reads obtained from DNA sequencing.
5. plasmid_name: The name used for labeling the plasmid assembly data or designing the name of the plasmid.
6. output_directory: The name of the folder or directory where the results, data, or files generated from an analysis are stored.
7. Type_of_run: To specify the running of the analysis pipeline, using “y” to refer to running the plasmid assembly function by using a reference sequence for assembly, or using “n” to refer to the running the plasmid assembly pipeline with the *de novo* assembly workflow.
8. Reference genome filename: The name of the reference file in FASTA format (.fasta), which is required only if the “type_of_run” is set to “y”; otherwise, it should be left empty.

The script iterates through each line of the text file, extracting the relevant information necessary for processing. Depending on the value specified in the “Type_of_run” column, the script follows one of two designed functions: The first function (Type_of_run = y) was designed for plasmid sequencing with a reference sequence. It uses a reference sequence for mapping. The mapping process includes the following steps: 1) Quality trimming of the reads by using fastp (Chen et al., 2018); 2) the alignment of reads to the reference using Bowtie2 (Langmead and Salzberg 2012); 3) the resulting SAM file is converted to the sorted BAM format (.bam) using samtool (Danecek et al., 2021); 4) the coverage information is generated to describe the average number of reads that aligned to a known reference at specific locations within the target reference sequence; 5) Low-coverage regions were identified using BEDtools (Quinlan and Hall, 2010) to filter these areas, as they may have higher error rates or may not be accurately represented in the assembled genome, being defined as ‘N' in the consensus sequence; 6) a VCF file is created to capture mutations in the mapped sequence using bcftool (Danecek et al., 2021); and 7) The consensus sequence is generated from the VCF file using bcftools and the custom python script. Finally, a report file in “.html” format is generated using an in-house python script. The report includes the coverage information, and a mutation plot generated from the VCF file. The consensus sequence serves as input for plasmid annotation, providing an annotation map in GenBank format (.gbk) which can be easily visualized and directly integrated into common plasmid design tools (e.g. Benchling, SnapGene). An interactive plasmid map in.html format is also generated using the pLannotate tool, providing a clear visualization in the final step of the annotation workflow (McGuffie and Barrick, 2021).

The second function (Type_of_run = n) was generated for plasmid assembly through the *de novo* assembly of short sequencing reads. It was designed to streamline data analysis without a reference sequence. The first step of the function involves applying fastp (Chen et al., 2018) to assess the quality of the input files to eliminate low-quality reads as the first function. The *de novo* assembly of short sequencing reads was done using Unicycler tools (Wick et al., 2017). A Burrows–Wheeler Alignment tool (BWA; Li and Durbin, 2009) was used for mapping by aligning the trimmed read sequences against the assembly sequence results from Unicycler. Finally, the assembled sequence serves as an input for plasmid annotation. It provides an annotation map in a GenBank format (.gbk) as well as an interactive plasmid map in.html format using the pLannotate tool as the method in the final mapping step (McGuffie and Barrick, 2021).

The script continues by generating individual assembly reports for each process run and indicates the performance of specific files, such as consensus sequences or annotations. A summary of the entire assembly process, encompassing a visualization of the several types of runs and their corresponding outcomes. This HTML report includes relevant statistics and graphical representations that enable users to ascertain the success of the assembly process.

### Recombinant Tn5 preparation

The pA-Tn5 gene (Addgene plasmid #124601) (Kaya-Okur et al., 2019) was cloned into pET26b (Novagen) along with an N-terminal His10-tag. The plasmid was transformed into the *E. coli* strain BL21-AI (Invitrogen) for recombinant pA-Tn5 expression. Bacteria containing the plasmid were pre-cultured in a medium containing 25 μg/mL kanamycin. When the OD_600_ reached 0.6, 0.2% (w/v) arabinose was added to induce protein expression at 18 °C overnight. Cell pellets were resuspended in buffer A [50 mM Tris-HCl, pH 7.5, 800 mM NaCl, 1 mM MgCl_2_, 1 mM DTT, 10% (v/v) glycerol, and 20 mM imidazole] with 0.5 mg/mL lysozyme and 0.1 mg/mL DNase I, then disrupted by sonication. The supernatant was loaded onto a 1 mL Ni-NTA Superflow column pre-equilibrated with buffer A. After loading the sample, the column was washed with buffer B [50 mM Tris-HCl, pH 7.5, 800 mM NaCl, 1 mM MgCl_2_, 1 mM DTT, 10% (v/v) glycerol, and 125 mM imidazole], and the recombinant His10-pA-Tn5 was eluted with 250 mM imidazole. The eluted sample was loaded onto a HiLoad 16/600 Superdex 200 pg column (Cytiva) in buffer C [50 mM Tris-HCl, pH 7.5, 800 mM NaCl, 0.2 mM EDTA, 2 mM DTT, 10% (v/v) glycerol]. The dimer of pA-Tn5 fractions was pooled and concentrated by ultrafiltration to a final concentration of 60 μM and stored at −80 °C until further use.

### *In vitro* reconstruction of Tn5 oligo complex

Preassembled Tn5 was prepared by following the protocols using Diagenode and a previous study (Picelli et al., 2014) with some modifications. Tagmentase (Tn5 transposase) - unloaded (Diagenode Cat# C01070010-10) was purchased from Diagenode and recombinant Tn5 protein was prepared in-house. Oligonucleotides (Tn5MErev, 5′-[phos] CTG TCT​CTT​ATA​CAC​ATC​T-3′; Tn5ME-A, 5′- TCG​TCG​GCA​GCG​TCA​GAT​GTG​TAT​AAG​AGA​CAG-3′; and Tn5ME- B, 5′-GTC​TCG​TGG​GCT​CGG​AGA​TGT​GTA​TAA​GAG​ACA​G-3′) were dissolved in TE buffer to prepare 100 μM stocks. Next, 1 μL of 10x annealing buffer (400 mM Tris-HCl, pH8.0, 500 mM NaCl) and 4.5 μL of 100 μM Tn5MErev was mixed with 4.5 μL of 100 μM Tn5ME-A or B and annealed using the following thermal cycler program: 95 °C for 5 min, gradually cooling (−0.1 °C/s), 65 °C for 5 min, gradually cooling to 4 °C. The annealed oligos were mixed in one tube. An equal volume of annealed oligonucleotide mixture and Tn5 (18.7 μM) were mixed and incubated at 23 °C for 30 min to assemble Tn5 and the annealed oligonucleotides. The preassembled Tn5 was diluted 10 times with 50% glycerol and stored at −20 °C.

### Library preparation for sequencing

The Tn5 reaction solution was prepared by mixing 3.65 μL of water, 1.1 µL of 5x TAPS-DMF buffer (50 mM TAPS-NaOH, pH8.5, 25mM MgCl_2_, and 40% DMF), 0.25 μL of preassembled Tn5, and 0.5 μL of plasmid. The Tn5-based tagmentation reaction was performed by incubating at 55 °C for 7 min. After adding 1.25 μL of 0.2% SDS, the tubes were incubated at 55 °C for 7 min. The PCR reaction consisted of 5 μL of 2x KOD One master mix (TOYOBO), 4 μL of water, 0.5 μL of Tn5 reaction product, and 0.5 μL of illumina combinatorial dual index primer mixture (5′-AATGATACGGCGACCACCGAGATCTACACNNNNNNNNTCGTCGGCAGCGTC-3′ and 5′-CAAGCAGAAGACGGCATACGAGATNNNNNNNNGTCTCGTGGGCTCGG-3′, where NNNNNNNN means appropriate index). The PCR reaction was performed at 98 °C for 2 min, followed by 30 cycles at 98 °C for 10 s, 55 °C for 5 s, and 68 °C for 5 s. An equal volume of the PCR products was mixed in a single tube. Amplicons were separated by electrophoresis (100 V, 30–40 min) on a 3% agarose gel and 250–500 bp fragments were collected by gel purification. The concentration of the purified DNA libraries was measured using a Qubit3.0 fluorometer (Thermo Fisher Scientific) and diluted to 50 pM based on our previous report (Yokomori et al., 2022). The 50 pM library was mixed with a 20%–30% volume of 50 pM PhiX (Illumina) and sequenced using an iSeq100 (Illumina) in 150 bp-PE mode.

### Plasmid sequencing of *E. coli* lysates

A single colony was suspended in 10 μL water and lysed by incubating at 95 °C for 10 min. After vortexing, the pellet was collected by centrifugation and 5 μL of the supernatant was removed from the remaining suspension. The *E. coli* genome was digested in the supernatant and the suspension with 0–10 U of exonuclease V (RecBCD) (New England Biolabs). The reaction was carried out in a 10-μL volume at 37 °C for 1 h. EDTA (10 mM in final concentration) was added and ExoV was heat inactivated at 70 °C for 30 min. The Tn5-based tagmentation reaction was done using 0.5 μL of the reaction product as indicated above.

### Reproducibility

The installation guide of AistSeq software is available as Supplementary Data 1. A frozen version of the analysis script is available as a self-contained environment in a Docker hub (https://hub.docker.com/r/pgrrg/aistseq_analysis). The script used for the data analyses and source data is available on Github (https://github.com/aist-pgrrg/aistseq). The workflow operates reliably on systems equipped with at least four CPU cores and 8 GB of RAM, although higher-performance hardware substantially improves execution speed. All analyses in this study were performed on an Apple MacBook Pro with an Apple M4 Pro system-on-chip, featuring a 14-core CPU with 10 performance cores (base ∼4.0 GHz, up to 4.5 GHz) and 4 efficiency cores (up to 2.6 GHz). The system used in this study was configured with 48 GB unified memory and a 1 TB SSD, which provided sufficient resources for all processing steps. For typical workloads, 30–50 GB of free disk space was required for temporary assembly, alignment, and intermediate output files. The pipeline is fully compatible with Ubuntu/Linux (20.04 and 22.04 LTS) and macOS (Monterey or later), and can also be executed on Windows 10/11 via WSL2 with comparable performance.

## Data Availability

The datasets presented in this study can be found in online repositories. The names of the repository/repositories and accession number(s) can be found below: https://github.com/aist-pgrrg/aistseq.
