# Supplementary material for: AistSeq: An in-house easy-to-purify Tn5-based plasmid sequencing platform using a compact benchtop sequencer
Source: Front Bioeng Biotechnol. 2026 Jan 27;13:1673510. doi: 10.3389/fbioe.2025.1673510 (PMC12886386; doi:10.3389/fbioe.2025.1673510)
Supplement: Supplementary file 1 [file Presentation1.pptx]

## Slide 1
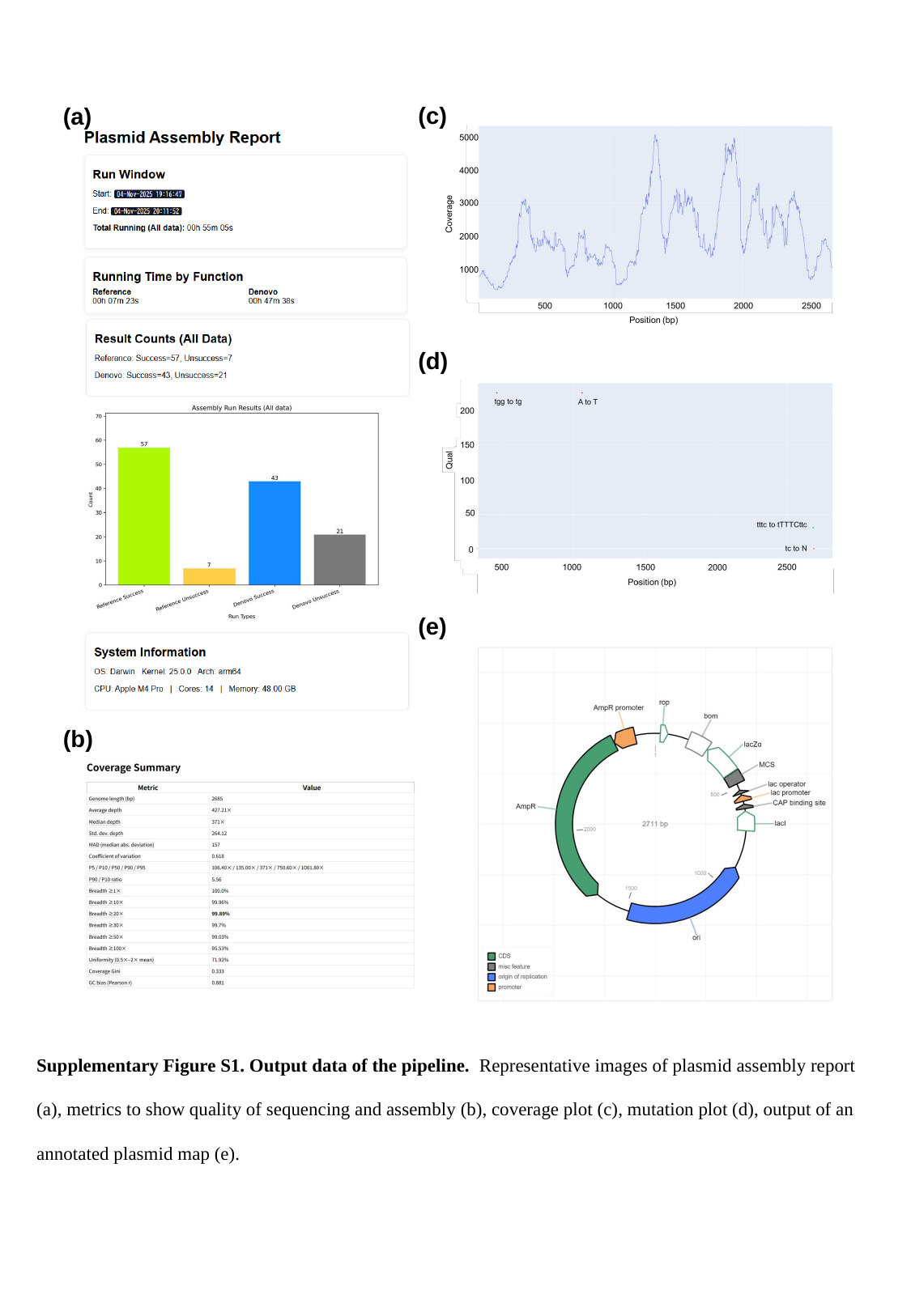

(c)
(a)
(d)
(e)
(b)
Supplementary Figure S1. Output data of the pipeline. Representative images of plasmid assembly report (a), metrics to show quality of sequencing and assembly (b), coverage plot (c), mutation plot (d), output of an annotated plasmid map (e).
