# Supplementary material for: AistSeq: An in-house easy-to-purify Tn5-based plasmid sequencing platform using a compact benchtop sequencer
Source: Front Bioeng Biotechnol. 2026 Jan 27;13:1673510. doi: 10.3389/fbioe.2025.1673510 (PMC12886386; doi:10.3389/fbioe.2025.1673510)
Supplement: Supplementary file 3 [file DataSheet1.docx]

## Supplementary Data S1. Installation, Deployment, and Execution Guide for the Plasmid Assembly Pipeline Installation and Execution

The pipeline can be executed on Linux, macOS, or Windows (via WSL2) and typically requires ≥4 CPU cores and ≥8–16 GB RAM. To maximize reproducibility and simplify deployment for non-expert users, we provide a frozen, version-stable Docker image that encapsulates all required dependencies (mappers, assemblers, and annotation utilities) in a self-contained environment (<https://hub.docker.com/r/pgrrg/aistseq_analysis>). The container is based on a Miniconda build and incorporates the same toolchain used for native execution. This approach eliminates manual tool installation, enabling the analysis to be executed uniformly across operating systems. For users who prefer native execution, the workflow can also be installed via Conda using two environments (`plasmid_analysis` and `plannotate`) that include bowtie2, bwa, samtools, bcftools, fastp, SPAdes, Unicycler, and pLannotate, as defined in the pipeline requirements. The primary entrypoint script is provided as Plasmid_assembly-2-Options-comand-V8_5.sh (hereafter referred to as plasmid_assembly.sh).

### ****Quick Start — Docker (Recommended for Non-Expert Users)****

Follow the steps below to run the pipeline using Docker on Windows. **Install Docker Desktop** and ensure it is running <https://docs.docker.com/engine/install/>

1. **Download the container image**:

docker pull jutapat/plasmid_assembly:latest

1. **Run the pipeline** (edit paths and filenames as needed):

**Windows (CMD) — mapping current folder to /data:**

docker run --rm -v <folder_on_your_computer>:/data jutapat/plasmid_assembly:latest bash plasmid_assembly.sh -i input_reads.zip -t sample_info.txt -o output_directory

**macOS/Linux — mapping current folder to /data:**

docker run –rm -v $(pwd):/data jutapat/plasmid_assembly:latest bash plasmid_assembly.sh -i input_reads.zip -t sample_info.txt -o output_directory

1. All results will be saved inside the mounted output directory on your computer (e.g., <folder_on_your_computer>/output_directory). The pipeline will generate a consensus FASTA, an annotated GenBank (.gbk), an interactive plasmid map (.html), and QC plots.

### Local (Via Conda) Installation — Advanced Users

Native installation mirrors the containerized toolchain using two Conda environments: plasmid_analysis.yml and plannotate.yml Ensure bowtie2 (and bowtie2-build), bwa, samtools, bcftools, bedtools, fastp, Unicycler, SPAdes (spades.py), pLannotate, and Python dependencies (pandas, numpy, matplotlib, scipy, statsmodels, pyfaidx) are available in PATH after environment activation. External resource paths referenced by the script (e.g., adapter FASTA, SPAdes entrypoint, bcftools directory, and reference database) should be checked and updated for your system before execution.

**Example run (native):**

bash Plasmid_assembly-2-Options-comand-V8_5.sh -i input_reads.zip -t sample_info.txt -o ./out_dir

### Troubleshooting

| **Problems** | **Reasons** | **Resolutions** |
| --- | --- | --- |
| Docker cannot access files (Permission denied / no files found) | The current drive/folder is not shared with Docker Desktop | Open Docker Desktop → Settings → Resources → File sharing; add your drive/folder. Re-run with -v <folder_on_your_computer>:/data |
| No output generated in the specified folder | Incorrect -i/-t paths or output directory not created | Confirm input filenames and paths; ensure -o output_directory is a valid relative name; check terminal for errors |
| Command not recognized (e.g., bash or script not found) | Container did not start or the working directory is not mapped | Use the full docker run command shown; verify the image is pulled; include -v <folder_on_your_computer>:/data and run from the folder containing inputs |
| Very slow performance | Insufficient CPU/RAM or competing heavy tasks | Close other applications; ensure ≥4 CPU cores and ≥8–16 GB RAM; consider SSD storage for input/output |
| File names with spaces cause errors | Unquoted paths in command-line | Surround file names with double quotes, e.g., -i "my reads.zip" -t "sample info.txt" |

- **Requirements for Running the Plasmid Pipeline**

This repository provides only the pipeline scripts. The following environments, tools, and data **must already be installed on the target machine.**

1. **Conda Environments Required**

- plasmid_analysis.yml
- plannotate.yml

**Tools (must be in** PATH **after activating the conda env)**

- bowtie2 (and bowtie2-build)
- bwa
- samtools
- bcftools
- bedtools
- fastp
- unicycler
- SPAdes (Python entrypoint: spades.py)
- plannotate
- Python with libraries: pandas, numpy, matplotlib, scipy, statsmodels, pyfaidx, (and others as needed)

1. **External Data / Paths Referenced in the Script** (Verify and update paths on the target machine if necessary)

- bcftools_path = $2 (or a valid bcftools bin path)
- default_reference_database = path/to/reference_database
- reference_database = $default_reference_database
- adapter_fasta = path/to/Desktop/Adapter/NexteraPE-PE.fa
- spades_path = path/to/SPAdes-3.15.5-Darwin/bin/spades.py
- bcftools_path = path/to/bcftools-1.18/bin
- **Tools Automatically Detected by the Script**
- bcftools, bedtools, bowtie2, bowtie2-build, bwa, fastp, plannotate, samtools, spades.py, unicycler

1. **How to Run on a New Machine**

- After, install the required tools and create both conda environments.
- Place your **input ZIP** and **TAB/INFO file** in a working directory.
- Execute the pipeline: bash Plasmid_assembly-2-Options-comand-V8_5.sh -i input.zip -t analysis_info.txt -o output_directory -DB reference_database_directory

## ****Argument Details****

| **Option** | **Long Form** | **Description** | **Example** |
| --- | --- | --- | --- |
| **-i** | --input-file | Path to the **ZIP file** containing FASTQ reads. | -i plasmid_reads.zip |
| **-t** | --text-file | Path to the **tab-delimited text file** containing sample information and run settings. | -t analysis_info.txt |
| **-o** | --output-dir | Path to the **output directory** where results will be written. Defaults to current directory. | -o ./results |
| **-DB** | --reference-database | Directory containing **reference FASTA files** for reference-guided assembly. | -DB ./reference_database |
| **-h** | --help | Displays help and example usage. | -h |

## ****Example Input Text File (****analysis_info.txt****)****

| Primary key | read1 | read2 | expected_reads | plasmid_name | output_directory | Type_of_run | Reference |
| --- | --- | --- | --- | --- | --- | --- | --- |
| 17 | PS-01A03_S3_L001_R1_001.fastq.gz | PS-01A03_S3_L001_R2_001.fastq.gz | 10000 | pTWIST_OsPYL1 | Game | **y** | **pENTR.fasta** |
| 18 | PS-01A03_S3_L001_R1_001.fastq.gz | PS-01A03_S3_L001_R2_001.fastq.gz | 10000 | pTWIST_OsPYL1 | Game | **n** | (blank) |

**Column meanings:**

1. **Primary key** – unique ID for each sample.
2. **read1 / read2** – paired-end FASTQ files inside the input ZIP.
3. **expected_reads** – optional expected number of reads (for QC).
4. **plasmid_name** – name used for output directory and labeling.
5. **output_directory** – subfolder name under main output directory.
6. **Type_of_run**:

- y = reference-guided assembly
- n = de novo assembly

1. **Reference** – name of reference FASTA (only required for y mode).
